# MetaDB a Data Processing Workflow in Untargeted MS-Based Metabolomics Experiments

**DOI:** 10.3389/fbioe.2014.00072

**Published:** 2014-12-16

**Authors:** Pietro Franceschi, Roman Mylonas, Nir Shahaf, Matthias Scholz, Panagiotis Arapitsas, Domenico Masuero, Georg Weingart, Silvia Carlin, Urska Vrhovsek, Fulvio Mattivi, Ron Wehrens

**Affiliations:** ^1^Research and Innovation Centre, Fondazione E. Mach, San Michele all’Adige, Trento, Italy; ^2^Institute of Plant Sciences, Faculty of Agriculture, The Hebrew University of Jerusalem, Rehovot, Israel

**Keywords:** metabolomics, ISA-Tab, pipeline, data analysis, LC-MS, GC-MS

## Abstract

Due to their sensitivity and speed, mass-spectrometry based analytical technologies are widely used to in metabolomics to characterize biological phenomena. To address issues like metadata organization, quality assessment, data processing, data storage, and, finally, submission to public repositories, bioinformatic pipelines of a non-interactive nature are often employed, complementing the interactive software used for initial inspection and visualization of the data. These pipelines often are created as open-source software allowing the complete and exhaustive documentation of each step, ensuring the reproducibility of the analysis of extensive and often expensive experiments. In this paper, we will review the major steps which constitute such a data processing pipeline, discussing them in the context of an open-source software for untargeted MS-based metabolomics experiments recently developed at our institute. The software has been developed by integrating our metaMS R package with a user-friendly web-based application written in Grails. MetaMS takes care of data pre-processing and annotation, while the interface deals with the creation of the sample lists, the organization of the data storage, and the generation of survey plots for quality assessment. Experimental and biological metadata are stored in the ISA-Tab format making the proposed pipeline fully integrated with the Metabolights framework.

## Introduction

The possibility of performing untargeted phenotyping and characterize in a semi-quantitative way complex phenomena has been driving the success of untargeted metabolomics over the last 10 years (Patti et al., 2012; Cho et al., 2014). Among the possible technological solutions, which can be used to perform untargeted metabolomics, mass-spectrometry based approaches are prominent, mainly due to their sensitivity and speed. In many cases, the mass spectrometers are coupled with chromatographic separation like in gas chromatography mass-spectrometry (GC-MS) or liquid chromatography mass-spectrometry (LC-MS) (Theodoridis et al., 2012). As an alternative, chromatography-free approaches – like direct infusion mass-spectrometry (DIMS) or flow injection (Fuhrer and Zamboni, 2015) – are also possible, in particular, to perform high-throughput screening, but in this paper, we will focus on the former.

A typical experiment deals with the analysis of tens to hundreds of samples, characterized by several thousands of metabolic features. These numbers are likely to increase further, considering the rapid evolution in automation and instrumental resolution and sensitivity. Technological development has a profound impact also on the operation of the analytical laboratory, where the sample/method management is often integrated into a laboratory information management system (LIMS). The explosion of available data and the growing need of large-scale high-throughput experiments require the development of automatic data storage, handling, and analysis solutions. These are of paramount importance for several practical and fundamental reasons:

- It is impossible to analyze manually the huge amount of data produced by complex and expensive holistic untargeted experiments.
- Reproducibility in the data analysis of huge datasets is an issue, in particular, if it is performed by interactive tools, which are not keeping track of the different steps. To prevent this issue, data processing routines should be stored and made available as a part of the publication process.
- Meta information on the sample should be stored and organized in open-source formats using codified terms and ontologies to allow for automatic information retrieval and data integration.
- Raw data are precious – in some cases, they are the results of analysis that cannot be replicated – so storage organization is of high importance.
- The use of open access repositories for raw data is expected to become a standard for the publication (Kirwan et al., 2014).

To fulfill the previous needs, it is necessary to introduce new tools and new concepts in all the steps of a metabolomics experiment, from sample collection to final data publication. Due to the complexity of the task and the conflicting needs of flexibility and robustness, the use of a single software package is often impractical and it is common to rely on pipelines.

In a pipeline, each separate node performs its task autonomously and the results are “piped” to the next step. This type of design is particularly efficient because each node can be developed using the most appropriate tool. Additionally, pipelines are flexible, because each element can be upgraded or tailored without tweaking the complete analysis workflow. For this reasons, several popular software tools like Knime (Warr, 2012)^1^ or Galaxy (Giardine et al., 2005; Blankenberg et al., 2010; Goecks et al., 2010)^2^ offer a high-level graphical interface for the design of data analysis pipelines. The attractiveness of the “pipeline” idea is also demonstrated by the development of web-based solution for the analysis data, like XCMSonline (Tautenhahn et al., 2012b)^3^, Metaboanalyst (Xia et al., 2009, 2012)^4^, or MetDat (Biswas et al., 2010) [see also Brown et al. (2005), Tohge and Fernie (2009)] for metabolomics. A web-based solution has the advantage of hiding to the final user all the details of the data processing machinery; unfortunately, it can be difficult to transfer the huge amounts of raw data produced by big -*omics* experiments. The algorithms running “behind the scenes” ensure good reproducibility of the data analysis process – at least in the short term. Some issues, however, could show up if one wants to reproduce the results of several years back, in particular, if they were obtained with a different version of the pipeline. New software releases indeed may include improved processing algorithms or new sets of default processing parameters. This implies that, for guaranteeing reproducibility, the version number of all the elements of the pipeline should be stored, and a repository for old versions must be available.

**Figure 1:**
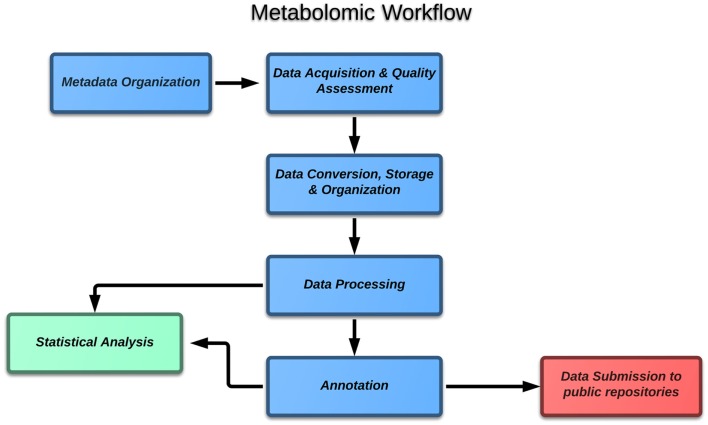
**Block diagram of the typical metabolomic experiment**.

If one considers the case of metabolomics, the block diagram (Figure 1) of the typical experiment includes the following major steps:

1. Metadata organization,
2. Data acquisition and quality assessment,
3. Data conversion, storage, and organization,
4. Data processing,
5. Annotation,
6. Statistical data analysis,
7. Data submission to public repositories.

The objective of this paper is to give a general account of each step from the point of view of the data analyst, highlighting central ideas and specific challenges. To make our survey more practical, each topic will also highlight some of the software solutions, which have been developed by the metabolomic community so far. The aim is not to present a comprehensive list, but instead to focus the key elements of the global picture. In the second part of the paper, we will also present the pipeline we have been developing at our institute, followed by an example application on a test experiment on grapevine leaves.

## Metabolomic Workflow in Details

In this section, each element of the block diagram is described in more details, referring to some of the more common strategies available for the community.

### Metadata

To be able to tackle relevant scientific questions, metabolomics have to adopt technologies and workflows, which can lead to “interoperable bioscience data” (Sansone et al., 2012). To fulfill this need, the experiments have to be well and consistently documented with a sufficient level of contextual information. For metabolomics, a strong effort toward standardization has been performed earlier by ArMet (Jenkins et al., 2004) and more recently by the Metabolomics Society with its Metabolomics Standard Initiative (Sansone et al., 2007). Different expert working groups have been identifying the minimal reporting standards for the biological context metadata, the chemical analysis, the data processing. More recently, standardization in metabolomics has become the core of the COSMOS initiative^5^ within the seventh Framework Program of the European Commission. The minimum reporting standards depends often on the type of study and should document the nature of the samples, the design of the experiment, and the details of the analytical pipeline (e.g., the LC-MS protocol). In the case of the biological source in plant studies, for example, one should include specie, genotype, organ, organ specification, cell type, subcellular location, and biosource amount. All this information should be provided relying as much as possible unique and well codified terms (ontologies). In practice, however, the use of ontology repositories could be non-trivial due the presence of synonyms or to the absence of terms describing a specific study or class of samples.

#### Proposed strategies

Considering the wealth of information needed to document properly each metabolomics experiments, a dedicated software solution can be of great help. In this way, human error can be reduced at minimum. Metadata should also be stored in open (and human readable) text formats, to allow their fast and automatic mining and interconversion. This type of information, then, can be used to automatically generate consistent sample names, avoiding mistyping or duplications.

SetupX (Scholz and Fiehn, 2006) is an example of software to store the details of a biological experiment into a database using publicly available taxonomic and ontology repositories, while the elements of the study design are employed to schedule and randomize data acquisitions.

A popular alternative to SetupX is ISAcreator. ISAcreator is a cross-platform java tool developed within the Isatools initiative (Rocca-Serra et al., 2010)^6^. ISAcreator guides the user in the process of inserting the metadata. In the background, they are stored in a specific text format (ISA-Tab), which has been designed to flexibly fit diverse scientific demands. Also, in this case, the description relies as much as possible on publicly available taxonomic and ontology repositories.

### Data acquisition and quality assessment

The data acquisition step is always managed by the instrument control software, which takes care of the sequential analysis of the samples contained in a “sample list.” The sample list can be generated manually inside the instrument software, or can be imported from an external file. Considering that the data analysis pipeline can produce the sequence file, this second option is the way to go when minimizing human intervention.

To obtain high-quality data, it is important to realize that the performance of the analytical method may change over time. This can profoundly affect the output of the experiment, in particular, in the case of long runs (instruments have to be periodically cleaned and calibrated, chromatographic columns are aging, etc.). For this reason, it is important to constantly monitor the quality of the analysis and to fully randomize the sample list to be sure that the factor of the study can be decoupled from any “analytical” perturbations. It is important to point out that analytical stability is not an absolute concept, but has to be judged in relation to the biological variability of the samples.

#### Proposed strategies

The commonly accepted practice to monitor drifts in the sample or in the analytical pipeline and to allow the equilibration of the analytical system is to include quality control (QC) samples (Sangster et al., 2006; Gika et al., 2014). QCs are also fundamental to correct for batch effects in large-scale profiling studies where it is impossible to include all the samples in the same analytical run (Dunn et al., 2011; Kirwan et al., 2013). A QC sample is something that is injected several times during the experiment so any changes in the results of its analysis are due to analytical drifts. Commonly, QC samples are either mixtures of chemical standards or pooled samples, similar to the ones under analysis (Dunn et al., 2011; Chen et al., 2014). The pooled QC has the advantage of being a more faithful representation of the “chemical” space spanned by the real samples. A good solution is to use QC samples of both types. How many of them should be included in the sequence has been already the subject of extensive investigation (Kamleh et al., 2012; Godzien et al., 2014).

In terms of quality assessment, it is common practice to monitor the signal of the internal standards added to the samples. A more general solution is to periodically process the acquired data and visualize them by a tool like principal component analysis (PCA) (Brown et al., 2005; Theodoridis et al., 2008). In the scoreplot, QC samples should cluster together and no sign of drift with the injection order should be visible. The relative standard deviation (RSD) of the intensity of the features across the QC samples is another important parameter that can be used to assess the quality of the analytical run. A distribution of the RSDs peaking below the 20% is considered a sign of good reproducibility (Dunn et al., 2011; Godzien et al., 2014).

At this level of the metabolomic workflow, the implementation of an automatic pipeline can give several advantages. First, it can easily create a fully randomized sample list with the appropriate number of QC samples using the files containing the experimental metadata. In this way, randomization is done by a computer and is bias-free; manual intervention is minimized and a coherent sample naming is ensured. Additionally, the pipeline can be easily used to generate QC plots to get an almost “real-time” feedback on the experimental run.

### Data conversion, storage, and organization

All mass spectrometers save raw data in proprietary formats. This lack of standardization is a strong limitation for the generation of “interoperable bioscience data” (Sansone et al., 2012). Many metabolomics laboratories are equipped with instruments of different models and different vendors and in such a situation data exchange and data comparison can easily become a nightmare. In an ideal world, raw data could be converted into open formats and then stored, in view of their analysis and submission to public repositories. Unfortunately, the real situation is not so simple: the conversion of raw data is possible, but an important part of the analytical information (configuration of the mass spectrometer and of the chromatograph, etc.) cannot be easily extracted. To avoid loss of precious information, then, it is commonplace to store the raw data in both formats, open and proprietary. This has to be taken into account when designing the data analysis pipeline and the storage space. To reduce space requirements data compression is advisable (Teleman et al., 2014), because open-source files can be non-compressed.

#### Proposed strategies

For the conversion of raw data in proprietary formats, several open-source standards are available. Among them, the common data format (CDF) is quite popular. Unfortunately, CDF files are not suitable to store multi-event MS experiments in a single file and are not designed to store spectral metadata (e.g., collision energy, precursor, etc.). More recently, XML-based solutions have been implemented [like mzXML (Pedrioli et al., 2004), mzML (Martens et al., 2011), etc.] and in this case multi-event experiments are supported.

As a general rule, data export is possible by using vendor-specific acquisition software, but unfortunately, a batch mode is not always supported, leading to long and tedious point-and-click sessions. As an alternative, the proteowizard (Kessner et al., 2008; Chambers et al., 2012) suite can be used for batch mode conversion, ideal for inclusion in a pipeline. The software is able to use the original vendor-specific libraries, which are available if the vendor data analysis software is installed. Unfortunately, not all types of files can be converted with the current version of the software: the most important exception is formed by the Waters QTOF RAW data for which the lock mass recalibration is not applied during conversion.

After conversion, the data have to be stored in a safe place and the best system to do that depends on the specific IT resources at hand. The cross-platform ISACreator software can be used to create a compressed archive by using information contained in the ISA-Tab file.

### Data processing

The term “data processing” commonly indicates the process of summarizing data into a matrix with the intensity of each experimental variable across all samples. This data matrix is then the starting point for the subsequent statistical analysis. In MS-based investigations, the experimental variables are mass-to-charge ratios (*m/z*), in the case of instruments without chromatographic separation, or tuples mass-to-charge ratio/retention time, for the more common LC/MS or GC/MS platforms. Some analytical platforms also implement ion mobility devices to separate the different ionic species, thus, adding a third “coordinate” to each variable. However, achieving a good chromatographic alignment across the various injections correcting for mass and retention time shifts is the first goal. This is done to make sure that one is comparing the same variable across all the samples. While the good stability of modern mass spectrometers guarantees a good reproducibility in the *m/z* dimension (accuracies below 3 ppm are common nowadays), the reproducibility in the chromatographic dimension can be an issue, in particular, in LC-MS. QC samples mentioned earlier can be used to monitor these types of problems.

Metabolomics aims at the comparison of the (relative) concentration of metabolites across samples. Unfortunately, the variables measured by MS-based technologies in most cases are not metabolites, but features. The problem is that there is no one-to-one correspondence between features and metabolites, because ionization is a complex process and several ions are generated from the same neutral molecule. In almost all cases, tens of different features in the data matrix represent each molecule. This redundancy further decreases the sample-to-variable ratio: cases where the number of samples is (much) smaller than the number of variables are called “fat” data matrices. In such a case, the statistical tools used for biomarker selection have to deal with the “multiplicity” problem, which actually limits the capacity of finding robust and consistent biomarkers, even from datasets with many samples (see later on).

To reduce the impact of this issue, it is necessary to take explicitly into account the relations among the different features, and to “group” them together. For each metabolite, this should lead to the reconstruction of its compound spectrum. These groups can be created by using chromatographic information (features coming from the same metabolite are co-eluting by definition) or by profiting from known chemical relations among the ions to identify isotopes and common adducts. At this level, it is also possible to take into account that the correlation of the different features across the different samples to increase the selectivity of the grouping step.

At the end of this phase, the most representative feature of each metabolite (or the pseudospectrum itself) can then be used to measure the relative concentration of each metabolite in the different samples, and to produce the “final” data matrix.

#### Proposed strategies

Data processing can be performed by using many different algorithms, and an extensive description of their specific steps is out of the scope of the present paper. The interested reader should refer to the documentation of each specific software package or to some comprehensive software review [e.g., Katajamaa and Oresic (2007), Castillo et al. (2011)]. As a general consideration, however, it is important to say that all the algorithms have advantages and limitations. For this reason, there is no ultimate solution, and – as always in metabolomics – it is necessary to find a good compromise. Vendor-specific softwares are provided by all the instrument manufacturers, and it is also possible to rely on cross-platform solutions like the open-source MZmine (Pluskal et al., 2010) and OpenMS (Sturm et al., 2008) or the freeware MetAlign (Lommen, 2009).

Some of these approaches have a graphical user interface; others can be implemented in graphical software suites or web-based interfaces to make them easily accessible to the lab scientist. As already discussed, however, graphical interactive tools are not ideal for pipelines, and scriptable solutions are to be preferred. Among these, a place of merit is held by the R [R Core Team (2014)] package XCMS (Smith et al., 2006). The development of the software started in 2006 and has gained momentum in the last years, also thanks to several ancillary R packages, which have been expanding its functionalities. The fact that it is written in R makes its integration with cutting-edge statistical packages trivial. Recently, the XCMS development team has been working on the integration of XCMS with ISA-Tab (González-Beltrán et al., 2014).

Also, feature grouping can be performed by different algorithms, e.g., the correlation-based R package CAMERA (Kuhl et al., 2011) and the clustering-based MSclust software (Tikunov et al., 2012).

### Annotation

The association of (groups of) experimental features to specific metabolites is commonly referred as “annotation” and it is, most likely, the biggest challenge for untargeted metabolomics experiments (Brown et al., 2009; Neumann and Böcker, 2010; Prasad et al., 2011; Dunn et al., 2013). As in the case of the meta information, the Metabolomic Standard Initiative recommends minimum reporting standards for annotation. The guidelines propose four different “confidence” levels of annotation. They go from “Identified Compounds” – for which the identification is confirmed by a comparison with the results of the analysis of a pure chemical standard – to “Unknown” – in the cases where nothing is known. The two intermediate levels are assigned to putatively annotated compounds or putatively annotated classes of compounds. For a certain identification, then, it is necessary to analyze a pure chemical standard in the same conditions as the samples (same chromatography, same MS method), complementing the mass information with an “orthogonal” property as the retention time.

All approaches that rely on the analysis of a set of chemical standards, however, suffer of two major drawbacks: first, in many cases it is necessary to re-measure the database of standards when the analytical method changes; second, not for all metabolites a commercial standard is available, in particular, for products of specialized metabolism. This second point is of particular importance for biological systems with high chemical diversity like plants or microorganisms. In addition, chemical standards can be very expensive.

The implementation of retention time prediction algorithms is a way to circumvent the first problem and reduce the experimental work. This approach gives good results in GC due to its higher separative potential and the much higher standardization of GC columns. Its extension to LC is the subject of important research efforts (Boswell et al., 2011; Creek et al., 2011; Hall et al., 2012; Stanstrup et al., 2013).

If a database containing chromatographic information is not available, it is also possible to rely on “pure” MS databases containing full scan information and fragmentation (MS/MS) spectra. This type of resource is often freely available online and there is an important effort by the scientific community to improve the quality, coverage, and standardization of online MS databases. Beyond this, annotation can be improved not only by taking advantage of many sources of external information like experimental metadata but also by the biological relations between the (partially) annotated metabolites (Creek and Barrett, 2014; Morreel et al., 2014).

At the end of all these steps, however, a big fraction of the features is still composed of unknown features, which – at the best – have been grouped together into putative pseudospectra. In this specific case, an interesting resource to go further can be represented by *in silico* fragmentation engines, which are used to propose MS spectra on the bases of the molecular properties of the molecules. The outcomes of these algorithms can be used as a helpful starting point to design further experimental assays aiming at a definitive chemical identification.

#### Proposed strategies

Highly informative annotation databases based on the analysis of pure chemical standards are developed in many laboratories, often using in-house or proprietary software. As in the case of raw data conversion, proprietary solutions can be easier to set up, but they have their limits as far as cross-platform/cross-laboratory portability is concerned.

In the case of open-source solutions, the R package metaMS (see later on) implements a strategy to generate in-house annotation databases both for LC and GC, based on the analysis of injections of (mixtures of) chemical reference standards.

In the case of pure MS databases, which are easier to compare across different instruments and different laboratories, it is possible to rely on rich online resources like “The Human Metabolome Database” (Wishart et al., 2007)^7^ and Mass Bank (Horai et al., 2010)^8^, the RIKEN ReSpect database for phytochemicals (Sawada et al., 2012), and the Metlin database (Tautenhahn et al., 2012a) to name a few. For a current list of available resources in the domain of plant biology, refer to Fukushima and Kusano (2013). Some of these databases can also be can be automatically queried to automatically confirm or propose metabolite annotation.

As far as “*in silico*” aided annotation, one could refer to the MetFrag fragmentation engine (Wolf et al., 2010), which can be incorporated into an R pipeline by using the (still experimental) MetFragR package^9^. Another interesting resource is the recently developed CFM-ID software package and web server (Allen et al., 2014), which offers an accessible and open software architecture. To improve annotation, *in silico* predictions can also be coupled with multistage MS^n^ data (Ridder et al., 2013).

As an alternative, an interesting approach is the one followed by the SIRIUS (Rasche et al., 2010) software, which uses the “similarity” of the fragmentation trees to assign unknowns to chemical and metabolic classes.

### Statistical data analysis

The development and application of statistical methods to metabolomics datasets is a full research field on its own and cannot be covered in this paper. The interested reader is referred to several review papers [e.g., Hendriks et al. (2011)]. In this context, however, it is useful to highlight some general properties of untargeted metabolomic datasets, which make their statistical analysis particularly challenging.

Raw metabolomics data are often characterized by “unwanted” variability of biological and technological nature. To try to account for these effects, it is necessary to perform sample normalization (De Livera et al., 2012) and this step should not interfere with the statistical analysis. Batch-to-batch variability is a particular case of technical variation, which is particularly important for large-scale studies. As already mentioned, QC samples can be used to minimize the impact of this phenomenon (Dunn et al., 2011; Kirwan et al., 2013).

From a more fundamental point of view, untargeted metabolomics experiments almost invariably result in “fat” data matrices where the number of variables by far exceeds the number of samples, and this tendency is aggravated by the steady increase in sensitivity of the new analytical platforms. In terms of statistical analysis and biomarker selection, these types of data are particularly challenging due to multiplicity issues (Franceschi et al., 2013). In addition, the features are often not independent and highly correlated, as a result of the ionization process itself, but also because of biological relations among the various metabolites. Even though an efficient annotation process would transform a feature-based data matrix into a compound/metabolite-based one, the result usually shows more metabolites than samples. For this reason, scientists often organize the different metabolites into higher level “networks” based on widespread chemical reactions (oxidation, reduction, …) or on prior metabolic knowledge (Creek and Barrett, 2014; Morreel et al., 2014).

### Data submission to public repositories

As a fundamental principle, the data supporting any scientific work must be made available to the research community to allow independent evaluation of the results. This practice has the additional value of contributing to the incremental progress of science, since new evidence or new experiments can be integrated with the established body of knowledge.

As is already the case in many other -*omics* technologies, the availability of the raw data is going to become a prerequisite for publication. To be useful, raw data should be made available in open formats, accompanied by a set of standardized metadata and organized into coherent repositories, the philosophy behind open-source data analysis pipelines.

#### Proposed strategies

As far as metabolomics experiments are concerned, an important repository for public datasets is the Metabolights Project (Haug et al., 2012) at the European Bioinformatic Institute (EBI)^10^. Datasets can be submitted in ISA-Tab format and can be made publicly available for the research community. The use of ISA-Tab ensures a high-level of quality and standardization of the datasets. The need of high-quality data open access repositories for -*omics* data is also demonstrated by “Scientific Data” initiative of the Nature Publishing Group^11^. In that journal, MetaboLights is listed among the “Recommended data repositories” for metabolomics data.

## Software Tools

This section describes the pipeline developed at our institute (partially), addressing the aforementioned challenges.

### MetaDB

To store and organize metabolomics data, a web-based software platform called MetaDB was developed. MetaDB is designed to make the implementation of a robust metabolomic data management workflow in the routinary laboratory practice. For each experiment, the interface requires as input the metadata as ISA-Tab files prepared by ISAcreator. Metadata are then used to create the randomized MS acquisition sequence and to store the data at the end of the experiment. MetaDB acts also as a high-level interface to the metaMS R package, which is used for data processing and peak list generation (see the following section for further details).

To keep data organized and ensure confidentiality the software requires a login and stores data for each user separately. Spectra data, results, and metadata can be uploaded, and later searched and downloaded again. Due to these functionalities, MetaDB can be used as a simple LIMS system.

**Figure 2:**
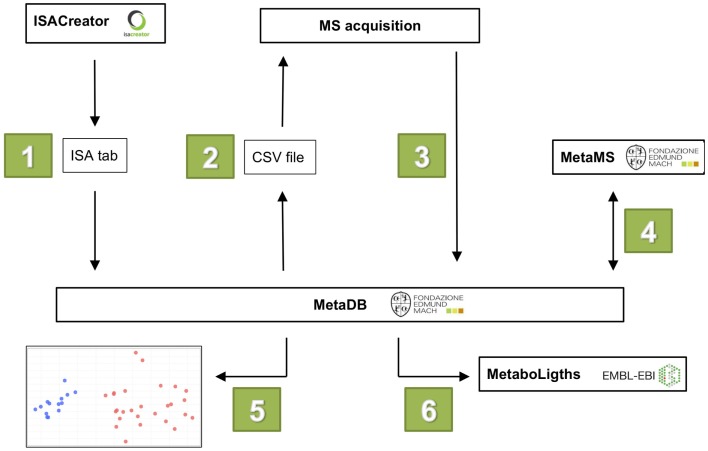
**The six steps in the MetaDB workflow**. (1) Metadata is uploaded to MetaDB using ISA-Tab formatted files. (2) A MS acquisition sequence is generated with randomized samples. (3) After MS data acquisition, raw and derived spectral data files are uploaded to MetaDB. (4) Data are processed using MetaMS. (5) Quality control of acquired data. (6) Data are prepared for storage and possibly for upload to public repositories such as MetaboLights.

The workflow of MetaDB consists of six different steps (see Figure 2):

1. Upload of metadata in ISA-Tab format.
2. Preparation of MS acquisition sequence, including sample randomization.
3. Upload of raw and derived spectral data files.
4. Data processing for feature alignment and detection with metaMS.
5. Visualization of data for quality assessment.
6. Preparation of data for upload to public repositories.

Steps 2–5 might have to be repeated to ensure the best analytical reproducibility. Between step 5 and 6, there may be additional data processing steps, currently not performed in the MetaDB workflow, but these can easily be included. To guarantee the reproducibility of each processing step, the software is returning also a log file (R_library_versions.log) with the version number of the R libraries loaded in background.

#### Description of metadata in ISA-Tab format

Metadata are inserted into MetaDB using the ISA-Tab file format. ISA-Tab was chosen for two different reasons. First, it is the format used by public data repositories such as MetaboLights. Second, convenient tools for creating and validating these files are already available (ISAcreator). The use of ISAcreator, also ensures the use of standardized terms for the description of the biological samples and of the analytical protocol.

#### Preparation of MS data acquisition sequence

After inserting metadata, an acquisition sequence is automatically generated. This sequence is based on a predefined pattern that includes the samples in randomized order, separated by blank injections (Blank) and QC samples. The nature of these QC samples depends on the experiment, but often a pooled sample is used, or, alternatively, a mixture of chemical standards (StdMix). The frequency and the number of these QC samples is a parameter stored in the MetaDB configuration settings.

Besides the important aspect of sample randomization, the use of an automatically generated acquisition sequence ensures a correct linking of the sample names with the spectral data files, preventing possible typing errors, which may occur during manual preparation of a sample list. From MetaDB, the acquisition sequence can be exported as a comma separated values (CSV) file and directly imported in the platform-specific MS Instrument control software.

#### Upload of raw and derived spectral data files

In many cases, the final acquisition sequence might differ from the one proposed by MetaDB. Typically, this happens because some of the samples or even parts of the acquisition have to be repeated, due to instrument failures or low quality acquisitions. To account for this common case, the actual sequence can be uploaded to MetaDB at the end of the experimental run. Also, at this level, the software checks the consistency between the acquisition names and the raw files present in the acquisition folder.

At the end of the acquisition run, the raw data are uploaded to MetaDB and automatically linked to their corresponding samples. MetaDB takes care of uploading the files on a network-attached storage (NAS) disk, connected with MetaDB. Alternatively, data can be compressed into a ZIP archive, which is uploaded through a web-interface. In order to be processed by the pipeline, the raw data have to be converted to open-source formats. As already discussed CDF, mzXML, or mzML files are commonly generated, either directly by the MS instrument control software or alternatively using external tools such as Proteowizard. After the conversion, those files are also uploaded to MetaDB in the same way as raw data. In a future version, direct transformation of raw files to derived spectral data files might be integrated into MetaDB.

#### MetaMS data processing for feature alignment and detection

Data processing is performed in background by the metaMS R package (see the following section). The user simply selects the set of processing parameters from a dropdown list, for clarity, all the parameters are associated to the name of the instrument used in the data acquisition phase. Additional sets of processing settings can be made available to the user as described in the metaDB manual. At this stage, it is possible to restrict the retention time considered in the data analysis. This can sometimes prevent sample misalignments. If a metaMS in-house database is available, it can be used to generate annotation information. For GC data, this is based on matching pseudospectra, for LC data individual peaks are matched.

This data analysis step can be repeated as often as necessary. After data processing is finished, the final peak table produced by metaMS can be either downloaded as a CSV file or as a binary RData file.

#### Quality control plots

After processing data with metaMS, MetaDB implements some basic plots, based on PCA and RSD calculation to make a quick survey of the analytical run and, if needed, suggests repeating some of the injections. As discussed in the previous sections, in a successful run, the cloud of “real” samples should be clearly separated from the QC injections and the blanks in the PCA scoreplot. This type of visualization allows the detection of sample drift, outliers or batch effects. Since PCA results are very much dependent on the scaling of the data, several types of scaling are available. As a further QC check, MetaDB displays a histogram showing the distribution of the RSDs of the intensities of the features across the QC samples.

To correct for small differences in the efficiency between the different samples within the analytical run, it is also possible to perform total ion current (TIC) normalization. Besides, the PCA and RSD plots, MetaDB also produces a plot showing the integrated TIC over each sample. This survey plot can reveal the presence of a global change in the sensitivity of the analytical platform.

#### Prepare data for storage

Once data processing is finished, the results and the raw data can be added to the ISA-Tab files containing the metadata, and stored either locally or in a web-based repository.

Source code of MetaDB, the installation description, and user manuals containing further technical information can be obtained from GitHub^12^. An Ubuntu 14.04 VirtualBox with preinstalled metaMS, web-framework and example data can be downloaded at https://drive.google.com/file/d/0B09xZzKu_n8yem9hdWY0VjQ2eDQ/view?usp=sharing.

MetaDB is written as a three-tier Java application using the Grails framework (version 2.2.3). MySQL (version 5.5) is used as a relational database. CSS layout and Javascript functionalities are based on the Twitter Bootstrap framework (version 3.0.2). The installation requires Java 1.7 and Tomcat 6.

All the Java code used for parsing the ISA-Tab files was taken from ISAcreator (version 1.7.5)^13^. ISA-Tab validation is achieved using the ISAcreator configuration (MetaboLightsConfig20130507) taken from MetaboLights (see text footnote 10).

### MetaMS

MetaMS (Wehrens et al., 2014) is an add-on to XCMS, available from the Bioconductor repository, developed specifically in the context of untargeted metabolomics providing facilities for building in-house databases of chemical standards. In the package, the key processing parameters are organized within a specific S4 class. With the associated methods, it is straightforward to define a new set of processing parameter optimized for the specific chromatographic and mass spectrometric conditions.

MetaMS can be used to process LC-MS and GC-MS analytical runs. For a detailed description of the GC-MS/LC-MS workflow, the reader can refer to the vignettes of metaMS. In summary, for LC-MS, the main part of the metaMS pipeline is similar to the XCMS pipeline, consisting of peak picking, grouping, and alignment. The additions from metaMS focus on improved annotation using in-house databases, an *m/z* and intensity-dependent mass accuracy window and an explicit definition of minimal support for annotation. The outcome is a matrix summarizing for all samples the intensities of the aligned peaks. The GC-MS pipeline in metaMS differs somewhat from the standard XCMS workflow, working on so-called pseudospectra rather than individual peaks. Here, the output is a relative intensity measure for chemical compounds rather than individual peaks. The compounds may be annotated (when there is a match with the database), or labeled as Unknowns.

## Illustrative Step-By-Step Workflow

In this section, we describe a complete metabolomic data analysis workflow on a test set of samples using our previously described pipeline.

### Sample description

Grape leaves were obtained from Regent and Phoenix varieties cultivated at experimental vineyards in Rattey (Mecklenburg West-Pomerania, North Germany) and San Michele all’Adige (Trentino, North Italy). Adult, undamaged, and healthy leaves from above the grape main zone were sampled at the development stage of the berries when the grape had ~70% of their size (July 2012). Four leaves were collected for each plant, from 10 different vines, in order to have 10 biological replicates for each combination of variety and country. The Italian samples were directly frozen and stored at −80°C, while the German grape samples were frozen under liquid nitrogen, packed in dry ice, and shipped to Italy within 24 h and stored at −80°C. The samples were analyzed by ultra performance liquid chromatography-time of flight mass-spectrometry (UPLC-QTOF-MS) with the analytical protocol described in the Supplementary Material. QC samples were obtained by mixing an aliquot of the powder obtained from the different samples.

### Metadata

An ISA-Tab file containing the metadata information of the experiment was created with ISACreator and is available at the MetaboLights website (MTBLS137). All necessary information concerning the experiment and the samples were entered as described in the MetaboLights user manual. Since at this stage the final order of the acquisition is not known, MS Assay Names were left free.

### MS data acquisition, conversion, and storage

The ISA-Tab file was uploaded to the metaDB web-interface to prepare the randomized sample list. The analytical sequence has the following pattern: at the beginning, there is 1 Blank, 1 chemical standard mix, followed by repetition of 4 samples, 1 StdMix until there are no samples left. The acquisition sequence was then exported as a comma separated version (CSV) file and directly imported into MassLynx (Waters Corporation) to set up the acquisition run. After the analytical run, the raw data were converted to CDF by using the Databridge conversion software included in Masslynx (Waters Corporation). The raw data and their converted version were uploaded to the final storage space through MetaDB.

### Data processing

Once extracted files were added (indicated by a blue “processed”), they were selected in the web-interface and analyzed using metaMS. All runs apart from the blank injections and the standard mixtures were selected. The data with retention times lower than 1 min and higher than 21 min were disregarded, annotation was not performed. The data were preprocessed with a set of parameters optimized for the specific chromatographic and instrumental conditions. The specific settings are included in the test virtual machine.

### Quality assessment

**Figure 3:**
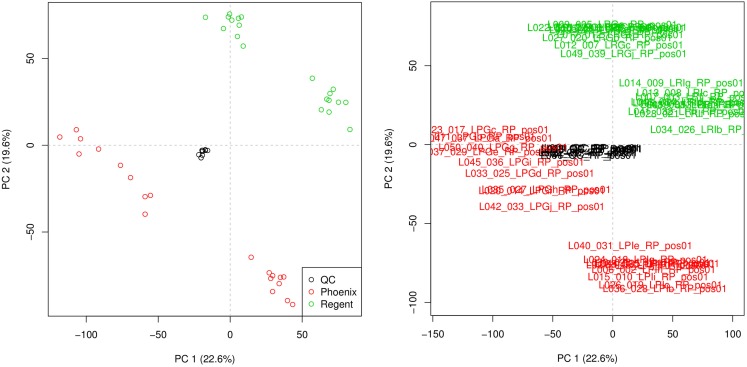
**Principal component analysis scoreplot produced by metaDB on the example dataset**. Different colors are used to identify the two different sample classes and the QCs. The different samples can be identified also by their sample names. This feature can be useful for the fast identification of critical samples.

Principal component analysis was used to check the quality of the injection. The resulting scoreplot is displayed in Figure 3. The points in the figure show the position of the different samples in the plane of higher variance. From the plot, the separation of the samples coming from the different grape varieties is clearly visible. One can see that the QCs constitute a tight cluster in the middle of the “real” samples. This behavior is the indication of a good analytical reproducibility because it shows that the variability due to the analytical platform (the spread of the black points) is much smaller than the biological variability between the samples.

**Figure 4:**
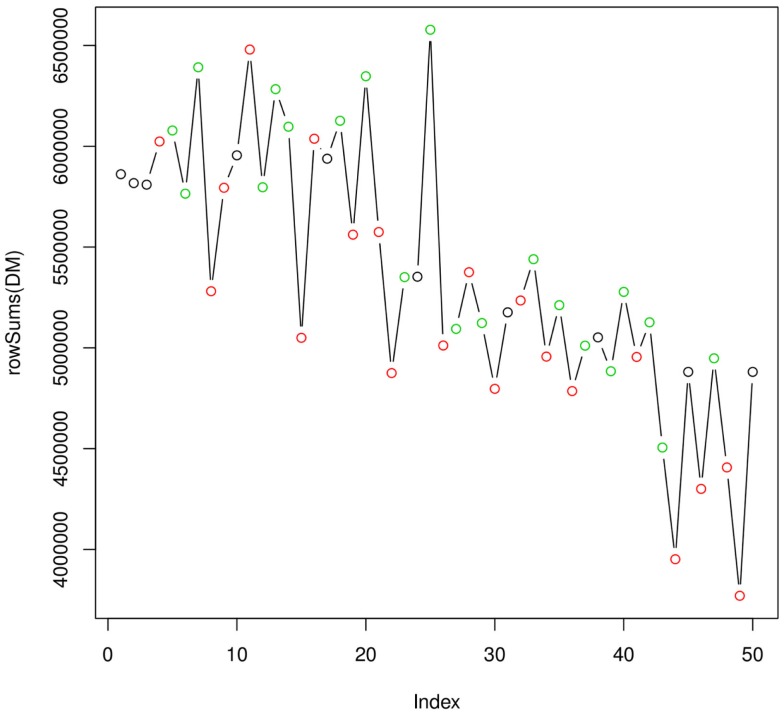
**Variation of the integral of the TIC over the analytical run**.

Figure 4 shows how the integral of the TIC varies over the full analytical run of 50 injections (~20 h). As expected, the efficiency of the instrument is decreasing as the ionization source gets less efficient. This plot does not indicate the presence of particularly critical injections, which would require the re-injection of some of the samples. Taken together, the two quality plots indicate a satisfactory analytical run. The experimental raw data can be then uploaded to metaDB, which will take care of their organization and long-term storage. At this stage, the peak table generated by metaMS can be downloaded and used for further statistical analysis.

## Conclusion

The use of robust and reproducible data analysis pipelines is a key prerequisite to exploit fully the analytical potential of untargeted metabolomics and *de facto* is mandatory to achieve scientifically reproducible results.

Pipelines can be realized in many different ways, but the use of high-level scripting languages like R or Python represent an ideal solution in term of robustness, speed of development, computational efficiency and reproducibility. In particular, they allow implementing in a modular and easy way cutting-edge bioinformatic tools for metabolomics.

To make these solution available and useful in the daily laboratory activity, the pipeline should be integrated with independent user-friendly skins, which take care of inserting them in the routinely analytical workflow, minimizing manual intervention as much as possible and maximizing the amount of high quality meta information.

The combined use of metaMS and MetaDB represent our solution to this growing need of standardization, both tools are open source and they have been designed to integrate smoothly with the EBI-EMBL Metabolight initiative.

## Future Work

MetaDB has been designed with the objective of matching the needs of researchers working in the laboratory while maintaining the minimal requirements of a robust metabolomics study. The inclusion of metaMS opens up all tools available in the XCMS/CAMERA software suites; in order to enhance user-friendliness and usability, processing parameters are grouped and stored in separate chunks, allowing the easy selection of “standard” settings for specific instruments. MetaDB is not designed to perform parameter optimization, which should be done manually at a lower level. It could, e.g., be useful to include in a separate template the possibility of optimizing the processing parameters, which could then be saved as metaMS settings object. The implementation of automatic algorithms to perform this optimization step (Brodsky et al., 2010) could also be possible.

A specific workflow to process the injections of the pure chemical standards and generate the database used for annotation, already part of the metaMS package, is planned for a forthcoming version of MetaDB.

## Conflict of Interest Statement

The authors declare that the research was conducted in the absence of any commercial or financial relationships that could be construed as a potential conflict of interest.

## Supplementary Material

The details of the analytical method used for the analysis of the test dataset are provided as supplementary material.The Supplementary Material for this article can be found online at http://www.frontiersin.org/Journal/10.3389/fbioe.2014.00072/abstract

## Footnotes

^1^http://www.knime.org/

^2^http://galaxyproject.org/

^3^http://xcmsonline.scripps.edu/

^4^http://www.metaboanalyst.ca/MetaboAnalyst/

^5^http://cosmos-fp7.eu/

^6^http://www.isa-tools.org/format.html

^7^http://www.hmdb.ca/

^8^http://www.massbank.jp/

^9^http://github.com/c-ruttkies/MetFragR

^10^http://www.ebi.ac.uk/metabolights/

^11^http://www.nature.com/sdata/

^12^http://github.com/rmylonas/MetaDB

^13^http://github.com/ISA-tools/ISAcreator

## References

[B1] Allen F. Pon A. Wilson M. Greiner R. Wishart D. (2014). CFM-ID: a web server for annotation, spectrum prediction and metabolite identification from tandem mass spectra. Nucleic Acids Res. 42, W94–W99. 10.1093/nar/gku436 24895432 PMC4086103

[B2] Biswas A. Mynampati K. C. Umashankar S. Reuben S. Parab G. Rao R. (2010). MetDAT: a modular and workflow-based free online pipeline for mass spectrometry data processing, analysis and interpretation. Bioinformatics 26, 2639–2640. 10.1093/bioinformatics/btq436 20702401

[B3] Blankenberg D. Kuster G. V. Coraor N. Ananda G. Lazarus R. Mangan M. (2010). Galaxy: a web-based genome analysis tool for experimentalists. Curr. Protoc. Mol. Biol. Chapter 19, Unit 19.10.1–21. 10.1002/0471142727.mb1910s89 20069535 PMC4264107

[B4] Boswell P. G. Schellenberg J. R. Carr P. W. Cohen J. D. Hegeman A. D. (2011). Easy and accurate high-performance liquid chromatography retention prediction with different gradients, flow rates, and instruments by back-calculation of gradient and flow rate profiles. J. Chromatogr. A 1218, 6742–6749. 10.1016/j.chroma.2011.07.070 21840007

[B5] Brodsky L. Moussaieff A. Shahaf N. Aharoni A. Rogachev I. (2010). Evaluation of peak picking quality in LC-MS metabolomics data. Anal. Chem. 82, 9177–9187. 10.1021/ac101216e 20977194

[B6] Brown M. Dunn W. B. Dobson P. Patel Y. Winder C. L. Francis-McIntyre S. (2009). Mass spectrometry tools and metabolite-specific databases for molecular identification in metabolomics. Analyst 134, 1322–1332. 10.1039/b901179j 19562197

[B7] Brown M. Dunn W. B. Ellis D. I. Goodacre R. Handl J. Knowles J. D. (2005). A metabolome pipeline: from concept to data to knowledge. Metabolomics 1, 39–51 10.1007/s11306-005-1106-4

[B8] Castillo S. Gopalacharyulu P. Yetukuri L. Orešič M. (2011). Algorithms and tools for the preprocessing of LC-MS metabolomics data. Chemometr. Intell. Lab. Syst. 108, 23–32. 10.1016/j.chemolab.2011.03.01025063004

[B9] Chambers M. C. Maclean B. Burke R. Amodei D. Ruderman D. L. Neumann S. (2012). A cross-platform toolkit for mass spectrometry and proteomics. Nat. Biotechnol. 30, 918–920 10.1038/nbt.2377 23051804 PMC3471674

[B10] Chen M. Rao R. S. P. Zhang Y. Zhong C. X. Thelen J. J. (2014). A modified data normalization method for GC-MS-based metabolomics to minimize batch variation. Springerplus 3, 1–7. 10.1186/2193-1801-3-439 25184108 PMC4149678

[B11] Cho K. Mahieu N. G. Johnson S. L. Patti G. J. (2014). After the feature presentation: technologies bridging untargeted metabolomics and biology. Curr. Opin. Biotechnol. 28, 143–148. 10.1016/j.copbio.2014.04.006 24816495 PMC4111999

[B12] Creek D. J. Barrett M. P. (2014). Determination of antiprotozoal drug mechanisms by metabolomics approaches. Parasitology 141, 83–92 10.1017/S0031182013000814 23734876 PMC3884841

[B13] Creek D. J. Jankevics A. Breitling R. Watson D. G. Barrett M. P. Burgess K. E. (2011). Toward global metabolomics analysis with hydrophilic interaction liquid chromatography-mass spectrometry: improved metabolite identification by retention time prediction. Anal. Chem. 83, 8703–8710. 10.1021/ac2021823 21928819

[B14] De Livera A. M. Dias D. A. De Souza D. Rupasinghe T. Pyke J. Tull D. (2012). Normalizing and integrating metabolomics data. Anal. Chem. 84, 10768–10776. 10.1021/ac302748b 23150939

[B15] Dunn W. B. Broadhurst D. Begley P. Zelena E. Francis-McIntyre S. Anderson N. (2011). Procedures for large-scale metabolic profiling of serum and plasma using gas chromatography and liquid chromatography coupled to mass spectrometry. Nat. Protoc. 6, 1060–1083. 10.1038/nprot.2011.335 21720319

[B16] Dunn W. B. Erban A. Weber R. J. Creek D. J. Brown M. Breitling R. (2013). Mass appeal: metabolite identification in mass spectrometry-focused untargeted metabolomics. Metabolomics 9, 44–66 10.1007/s11306-012-0434-4

[B17] Franceschi P. Giordan M. Wehrens R. (2013). Multiple comparisons in mass-spectrometry-based-omics technologies. Trends Analyt. Chem. 50, 11–21. 10.1016/j.trac.2013.04.01122438836

[B18] Fuhrer T. Zamboni N. (2015). High-throughput discovery metabolomics. Curr. Opin. Biotechnol. 31, 73–78 10.1016/j.copbio.2014.08.006 25197792

[B19] Fukushima A. Kusano M. (2013). Recent progress in the development of metabolome databases for plant systems biology. Front. Plant Sci. 4:73. 10.3389/fpls.2013.00073 23577015 PMC3616245

[B20] Giardine B. Riemer C. Hardison R. C. Burhans R. Elnitski L. Shah P. (2005). Galaxy: a platform for interactive large-scale genome analysis. Genome Res. 15, 1451–1455. 10.1101/gr.4086505 16169926 PMC1240089

[B21] Gika H. G. Theodoridis G. A. Plumb R. S. Wilson I. D. (2014). Current practice of liquid chromatography-mass spectrometry in metabolomics and metabonomics. J. Pharm. Biomed. Anal. 87, 12–25 10.1016/j.jpba.2013.06.032 23916607

[B22] Godzien J. Alonso-Herranz V. Barbas C. Armitage E. G. (2014). Controlling the quality of metabolomics data: new strategies to get the best out of the QC sample. Metabolomics 87, 1–11 10.1007/s11306-014-0712-4

[B23] Goecks J. Nekrutenko A. Taylor J. The Galaxy Team. (2010). Galaxy: a comprehensive approach for supporting accessible, reproducible, and transparent computational research in the life sciences. Genome Biol. 11, R86. 10.1186/gb-2010-11-8-r86 20738864 PMC2945788

[B24] González-Beltrán A. Neumann S. Maguire E. Sansone S. A. Rocca-Serra P. (2014). The Risa R/Bioconductor package: integrative data analysis from experimental metadata and back again. BMC Bioinformatics 15:S11. 10.1186/1471-2105-15-S1-S11 24564732 PMC4015122

[B25] Hall L. M. Hall L. H. Kertesz T. M. Hill D. W. Sharp T. R. Oblak E. Z. (2012). Development of Ecom50 and retention index models for nontargeted metabolomics: identification of 1, 3-dicyclohexylurea in human serum by HPLC/mass spectrometry. J. Chem. Inf. Model. 52, 1222–1237. 10.1021/ci300092s 22489687 PMC3376006

[B26] Haug K. Salek R. M. Conesa P. Hastings J. de Matos P. Rijnbeek M. (2012). MetaboLights – an open-access general-purpose repository for metabolomics studies and associated meta-data. Nucleic Acids Res. 41, D781–D786. 10.1093/nar/gks1004 23109552 PMC3531110

[B27] Hendriks M. M. Eeuwijk F. A. V. Jellema R. H. Westerhuis J. A. Reijmers T. H. Hoefsloot L. H. (2011). Data-processing strategies for metabolomics studies. Trends Analyt. Chem. 30, 1685–1698 10.1016/j.trac.2011.04.019

[B28] Horai H. Arita M. Kanaya S. Nihei Y. Ikeda T. Suwa K. (2010). MassBank: a public repository for sharing mass spectral data for life sciences. J. Mass Spectrom. 45, 703–714. 10.1002/jms.1777 20623627

[B29] Jenkins H. Hardy N. Beckmann M. Draper J. Smith A. R. Taylor J. (2004). A proposed framework for the description of plant metabolomics experiments and their results. Nat. Biotechnol. 22, 1601–1606. 10.1038/nbt1041 15583675

[B30] Kamleh M. A. Ebbels T. M. Spagou K. Masson P. Want E. J. (2012). Optimizing the use of quality control samples for signal drift correction in large-scale urine metabolic profiling studies. Anal. Chem. 84, 2670–2677. 10.1021/ac202733q 22264131

[B31] Katajamaa M. Oresic M. (2007). Data processing for mass spectrometry-based metabolomics. J. Chromatogr. A 1158, 318–328. 10.1016/j.chroma.2007.04.021 17466315

[B32] Kessner D. Chambers M. Burke R. Agus D. Mallick P. (2008). ProteoWizard: open source software for rapid proteomics tools development. Bioinformatics 24, 2534–2536. 10.1093/bioinformatics/btn323 18606607 PMC2732273

[B33] Kirwan J. A. Broadhurst D. I. Davidson R. L. Viant M. R. (2013). Characterising and correcting batch variation in an automated direct infusion mass spectrometry (DIMS) metabolomics workflow. Anal. Bioanal. Chem. 405, 5147–5157. 10.1007/s00216-013-6856-7 23455646

[B34] Kirwan J. A. Weber R. J. Broadhurst D. I. Viant M. R. (2014). Direct infusion mass spectrometry metabolomics dataset: a benchmark for data processing and quality control. Sci. Data 1 10.1038/sdata.2014.12 PMC4381748 25977770

[B35] Kuhl C. Tautenhahn R. Böttcher C. Larson T. R. Neumann S. (2011). CAMERA: an integrated strategy for compound spectra extraction and annotation of liquid chromatography/mass spectrometry data sets. Anal. Chem. 84, 283–289. 10.1021/ac202450g 22111785 PMC3658281

[B36] Lommen A. (2009). MetAlign: interface-driven, versatile metabolomics tool for hyphenated full-scan mass spectrometry data preprocessing. Anal. Chem. 81, 3079–3086. 10.1021/ac900036d 19301908

[B37] Martens L. Chambers M. Sturm M. Kessner D. Levander F. Shofstahl J. (2011). mzML – a community standard for mass spectrometry data. Mol. Cell Proteomics 10, R110–R000133. 10.1074/mcp.R110.000133 20716697 PMC3013463

[B38] Morreel K. Saeys Y. Dima O. Lu F. Van de Peer Y. Vanholme R. (2014). Systematic structural characterization of metabolites in *Arabidopsis* via candidate substrate-product pair networks. Plant Cell 26, 929–945. 10.1105/tpc.113.122242 24685999 PMC4001402

[B39] Neumann S. Böcker S. (2010). Computational mass spectrometry for metabolomics: identification of metabolites and small molecules. Anal. Bioanal. Chem. 398, 2779–2788. 10.1007/s00216-010-4142-5 20936272

[B40] Patti G. J. Yanes O. Siuzdak G. (2012). Innovation: metabolomics: the apogee of the omics trilogy. Nat. Rev. Mol. Cell Biol. 13, 263–269. 10.1038/nrm3314 22436749 PMC3682684

[B41] Pedrioli P. G. Eng J. K. Hubley R. Vogelzang M. Deutsch E. W. Raught B. (2004). A common open representation of mass spectrometry data and its application to proteomics research. Nat. Biotechnol. 22, 1459–1466. 10.1038/nbt1031 15529173

[B42] Pluskal T. Castillo S. Villar-Briones A. Orešič M. (2010). MZmine 2: modular framework for processing, visualizing, and analyzing mass spectrometry-based molecular profile data. BMC Bioinformatics 11:395. 10.1186/1471-2105-11-395 20650010 PMC2918584

[B43] Prasad B. Garg A. Takwani H. Singh S. (2011). Metabolite identification by liquid chromatography-mass spectrometry. Trends Analyt. Chem. 30, 360–387 10.1016/j.trac.2010.10.014

[B44] R Core Team. (2014). R: A Language and Environment for Statistical Computing. Vienna: R Foundation for Statistical Computing Available at: http://www.R-project.org/

[B45] Rasche F. Svatos A. Maddula R. K. Böttcher C. Böcker S. (2010). Computing fragmentation trees from tandem mass spectrometry data. Anal. Chem. 83, 1243–1251. 10.1021/ac101825k 21182243

[B46] Ridder L. van der Hooft J. J. Verhoeven S. de Vos R. C. Bino R. J. Vervoort J. (2013). Automatic chemical structure annotation of an LC-MS^n^ based metabolic profile from green tea. Anal. Chem. 85, 6033–6040. 10.1021/ac400861a 23662787

[B47] Rocca-Serra P. Brandizi M. Maguire E. Sklyar N. Taylor C. Begley K. (2010). ISA software suite: supporting standards-compliant experimental annotation and enabling curation at the community level. Bioinformatics 26, 2354–2356. 10.1093/bioinformatics/btq415 20679334 PMC2935443

[B48] Sangster T. Major H. Plumb R. Wilson A. J. Wilson I. D. (2006). A pragmatic and readily implemented quality control strategy for HPLC-MS and GC-MS-based metabonomic analysis. Analyst 131, 1075–1078. 10.1039/b604498k 17003852

[B49] Sansone S. A. Rocca-Serra P. Field D. Maguire E. Taylor C. Hofmann O. (2012). Toward interoperable bioscience data. Nat. Genet. 44, 121–126. 10.1038/ng.1054 22281772 PMC3428019

[B50] Sansone S. A. Schober D. Atherton H. J. Fiehn O. Jenkins H. Rocca-Serra P. (2007). Metabolomics standards initiative: ontology working group work in progress. Metabolomics 3, 249–256 10.1007/s11306-007-0069-z

[B51] Sawada Y. Nakabayashi R. Yamada Y. Suzuki M. Sato M. Sakata A. (2012). RIKEN tandem mass spectral database (ReSpect) for phytochemicals: a plant-specific MS/MS-based data resource and database. Phytochemistry 82, 38–45. 10.1016/j.phytochem.2012.07.007 22867903

[B52] Scholz M. Fiehn O. (2006). “SetupX – a public study design database for metabolomic projects,” in Pacific Symposium on Biocomputing (Grand Wailea, Maui, Hawaii: World Scientific Press), 169–180. 17990490

[B53] Smith C. A. Want E. J. O’Maille G. Abagyan R. Siuzdak G. (2006). XCMS: processing mass spectrometry data for metabolite profiling using nonlinear peak alignment, matching, and identification. Anal. Chem. 78, 779–787. 10.1021/ac051437y 16448051

[B54] Stanstrup J. Gerlich M. Dragsted L. O. Neumann S. (2013). Metabolite profiling and beyond: approaches for the rapid processing and annotation of human blood serum mass spectrometry data. Anal. Bioanal. Chem. 405, 5037–5048. 10.1007/s00216-013-6954-6 23615935

[B55] Sturm M. Bertsch A. Gröpl C. Hildebrandt A. Hussong R. Lange E. (2008). OpenMS – an open-source software framework for mass spectrometry. BMC Bioinformatics 9:163. 10.1186/1471-2105-9-163 18366760 PMC2311306

[B56] Tautenhahn R. Cho K. Uritboonthai W. Zhu Z. Patti G. J. Siuzdak G. (2012a). An accelerated workflow for untargeted metabolomics using the METLIN database. Nat. Biotechnol. 30, 826–828 10.1038/nbt.2348 22965049 PMC3666346

[B57] Tautenhahn R. Patti G. J. Rinehart D. Siuzdak G. (2012b). XCMS online: a web-based platform to process untargeted metabolomic data. Anal. Chem. 84, 5035–5039. 10.1021/ac300698c 22533540 PMC3703953

[B58] Teleman J. Dowsey A. W. Gonzalez-Galarza F. F. Perkins S. Pratt B. Röst H. L. (2014). Numerical compression schemes for proteomics mass spectrometry data. Mol. Cell. Proteomics 13, 1537–1542. 10.1074/mcp.O114.037879 24677029 PMC4047472

[B59] Theodoridis G. Gika H. G. Wilson I. D. (2008). LC-MS-based methodology for global metabolite profiling in metabonomics/metabolomics. Trends Analyt. Chem. 27, 251–260 10.1016/j.trac.2008.01.008

[B60] Theodoridis G. A. Gika H. G. Want E. J. Wilson I. D. (2012). Liquid chromatography-mass spectrometry based global metabolite profiling: a review. Anal. Chim. Acta 711, 7–16. 10.1016/j.aca.2011.09.042 22152789

[B61] Tikunov Y. M. Laptenok S. Hall R. D. Bovy A. de Vos R. C. H. (2012). MSClust: a tool for unsupervised mass spectra extraction of chromatography-mass spectrometry ion-wise aligned data. Metabolomics 8, 714–718. 10.1007/s11306-011-0368-2 22833709 PMC3397229

[B62] Tohge T. Fernie A. R. (2009). Web-based resources for mass-spectrometry-based metabolomics: a user’s guide. Phytochemistry 70, 450–456. 10.1016/j.phytochem.2009.02.004 19285697

[B63] Warr W. A. (2012). Scientific workflow systems: pipeline pilot and KNIME. J. Comput. Aided Mol. Des. 26, 801–804 10.1007/s10822-012-9577-7 22644661 PMC3414708

[B64] Wehrens R. Weingart G. Mattivi F. (2014). metaMS: an open-source pipeline for GC-MS-based untargeted metabolomics. J. Chromatogr. B 966, 109–116. 10.1016/j.jchromb.2014.02.051 24656939

[B65] Wishart D. S. Tzur D. Knox C. Eisner R. Guo A. C. Young N. (2007). HMDB: the human metabolome database. Nucleic Acids Res. 35(Suppl. 1), D521–D526. 10.1093/nar/gkl923 17202168 PMC1899095

[B66] Wolf S. Schmidt S. Müller-Hannemann M. Neumann S. (2010). In silico fragmentation for computer assisted identification of metabolite mass spectra. BMC Bioinformatics 11:148. 10.1186/1471-2105-11-148 20307295 PMC2853470

[B67] Xia J. Mandal R. Sinelnikov I. V. Broadhurst D. Wishart D. S. (2012). MetaboAnalyst 2.0 – a comprehensive server for metabolomic data analysis. Nucleic Acids Res. 40, W127–W133. 10.1093/nar/gks374 22553367 PMC3394314

[B68] Xia J. Psychogios N. Young N. Wishart D. S. (2009). MetaboAnalyst: a web server for metabolomic data analysis and interpretation. Nucleic Acids Res. 37(Suppl. 2), W652–W660. 10.1093/nar/gkp356 19429898 PMC2703878

