# Supplementary material for: MetaDB a Data Processing Workflow in Untargeted MS-Based Metabolomics Experiments
Source: Front Bioeng Biotechnol. 2014 Dec 16;2:72. doi: 10.3389/fbioe.2014.00072 (PMC4267269; doi:10.3389/fbioe.2014.00072)
Supplement: Supplementary file 1 [file Presentation_1.PDF]

Supplementary Material

Analytical conditions for the test dataset

HPLC

A Waters Acquity UPLC (Waters, Manchester, UK) controlled by MassLynx 4.1 was used. All samples were analysed on a reversed phase (RP) ACQUITY UPLC 1.8 µm 2.1 x 150 mm HSS T3 column (Waters) protected with an Acquity UPLC® BEH HSS T3 1.8 µm, 2.1 x 5 mm precolumn (Waters), at 40 oC and under a mobile phase flow rate of 0.28 mL/min. Water was used as weak eluting solvent (A) and methanol as strong eluting solvent (B); formic acid 0.1% v/v was used as additive in both eluents. The multistep linear gradient used was as follows: 0-1 min, 100% A isocratic; 1-3 min, 100-90 % A; 3-18 min, 90-60 % A; 18-21 min, 60-0 % A; 21-25.5 min, 0 % A isocratic; 25.5-25.6 min, 0-100 % A; 25.6-28 min 100% isocratic. Injection volume was 10 µL, and the samples were kept at 4 ºC throughout the analysis.

LC-MS

The Waters Acquity UPLC was coupled via an electrospray ionization (ESI) interface to a Synapt HDMS QTOF MS (Waters, Manchester, UK) operating in W-mode and controlled by MassLynx 4.1 was used. Mass spectrometric data were collected by separate runs in positive and negative ESI mode over a mass range of 50 to 2000 amu with scan duration of 0.3 s in centroid mode. The transfer collision energy and trap collision energy were set at 6 V and 4 V. The source parameters were set as follows: capillary 3 kV for positive scan and 2.5 kV for negative scan, sampling cone 25 V, extraction cone 3V, source temperature 150 ºC, desolvation temperature 500 ºC, desolvation gas flow 1000 L/h and nebulizer gas 50 L/h. External calibration of the instrument was performed at the beginning of each batch of analysis by direct infusion of a sodium formate solution (10% formic acid/0.1 M NaOH/Acetonitrile at a ratio of 1/1/8), controlling the mass accuracy from 40 to 2000 m/z (less than 5 ppm) and mass resolution (over 14000 FWHM). LockMass calibration was applied using a solution of leucine enkephaline (0.5 mg/L, m/z 554.2620 for negative ion mode) at 0.1 mL/min.
